# A Novel Hemagglutinin with Antiproliferative Activity against Tumor Cells from the Hallucinogenic Mushroom *Boletus speciosus*

**DOI:** 10.1155/2014/340467

**Published:** 2014-05-29

**Authors:** Jian Sun, Tzi-Bun Ng, Hexiang Wang, Guoqing Zhang

**Affiliations:** ^1^Key Laboratory of Urban Agriculture (North) of Ministry of Agriculture, College of Biological Sciences and Engineering, Beijing University of Agriculture, Beijing 102206, China; ^2^State Key Laboratory for Agrobiotechnology and Department of Microbiology, China Agricultural University, Beijing 100193, China; ^3^School of Biomedical Sciences, Faculty of Medicine, The Chinese University of Hong Kong, Shatin, New Territories, Hong Kong

## Abstract

Little was known about bioactive compounds from the hallucinogenic mushroom *Boletus speciosus*. In the present study, a hemagglutinin (BSH, *B. speciosus* hemagglutinin) was isolated from its fruiting bodies and enzymatic properties were also tested. The chromatographic procedure utilized comprised anion exchange chromatography on Q-Sepharose, cation exchange chromatography on CM-Cellulose, cation exchange chromatography on SP-Sepharose, and gel filtration by FPLC on Superdex 75. The hemagglutinin was a homodimer which was estimated to be approximately 31 kDa in size. The activity of BSH was stable up to 60°C, while there was a precipitous drop in activity when the temperature was elevated to 70°C. BSH retained 25% hemagglutinating activity when exposed to 100 mM NaOH and 25 mM HCl. The activity was potently inhibited by 1.25 mM Hg^2+^ and slightly inhibited by Fe^2+^, Ca^2+^, and Pb^2+^. None of the sugars tested showed inhibition towards BSH. Its hemagglutinating activity towards human erythrocytes type A, type B, and type AB was higher than type O. The hemagglutinin showed antiproliferative activity towards hepatoma Hep G2 cells and mouse lymphocytic leukemia cells (L1210) *in vitro*, with IC_50_ of 4.7 **μ**M and 7.0 **μ**M, respectively. It also exhibited HIV-1 reverse transcriptase inhibitory activity with an IC_50_ of 7.1 **μ**M.

## 1. Introduction

Lectins and hemagglutinins are nonimmune proteins or glycoproteins that exhibit specific binding to carbohydrates on the cell surface. They can agglutinate cells through sugar-specific binding sites for polysaccharides and glycoconjugates [1]. Lectins were first identified in plants and are now known to be distributed widely throughout the nature, including plants, animals, fungi, bacteria, and viruses [2]. In plants, they are mainly storage proteins and serve in defence against insects or fungi [3]. Lectins or hemagglutinins are important receptors in animals, such as human C-type lectin langerin [4] and rat liver asialoglycoprotein receptor [5]. In mushrooms, lectins play a pivotal role in dormancy, growth, morphogenesis, morphological changes consequent on parasitic infections, and molecular recognition during the early stages of mycorrhization [3].

Mushrooms of the* Boletus *genus have been known since ancient times for their delicious taste as edible wild food. However, some of them are toxic. There are only several reports about bioactive substance such as lectins [6, 7], ribonucleases [8], and toxic proteins [9] from this genus.* Boletus speciosus* is a rare wild hallucinogenic mushroom and can cause “lilliputian hallucination” when cooked in a wrong way or eaten too much. The stipe of the mushroom is yellow and becomes blue quickly after bruise. That is why locals of Southwest China call the mushroom “Jian Shou Qing” which means the mushroom changes to blue when touched. The toxicity of the mushroom is neuropsychiatric, and the patient can see little joyous creatures jumping and dancing around. The toxin was defined as hallucinogens but not isolated up to now [10]. On the other hand, the mushroom is considered as a delicious mushroom by local people since the poisoning symptoms vanished after being boiled up.

There is dearth information pertaining to bioactive substances of* B. speciosus*. The present investigation aimed to isolate and characterize a hemagglutinin from the fruiting body of* B. speciosus* and compare its characteristics with other known lectins or hemagglutinins from* Boletus* genus such as* Boletus edulis *lectin [11].

## 2. Materials and Methods

### 2.1. Isolation of Hemagglutinin

Ion exchange chromatography of dried* B. speciosus* fruiting bodies (20 g) was carried out using a 10 × 100 cm column of Q-Sepharose (GE Healthcare). The extract was first homogenized in 0.15 M NaCl (10 mL per gram) and extracted overnight at 4°C. Then the homogenate was centrifuged at 12,000 rpm for 30 minutes at 4°C. The supernatant was collected and (NH_4_)_2_SO_4_ was added to the supernatant until 80% saturation is reached. The mixture was left at 4°C for 8 hours before centrifugation at 10,000 rpm for 25 min. The precipitate was redissolved and dialyzed to remove (NH_4_)_2_SO_4_ before applying to a Q-Sepharose column in 10 mM Tris-HCl buffer (pH 8.0). After removal of the unadsorbed fraction Q1, adsorbed proteins were desorbed stepwise with 50 mM NaCl in the Tris-HCl buffer to yield fraction Q2 and then with 300 mM and 1 M NaCl in the Tris-HCl buffer to yield fractions Q3 and Q4. Fraction Q3 with hemagglutinating activity was then subjected to ion exchange chromatography on a 2.5 × 30 cm column of CM-Cellulose (Sigma) in 10 mM sodium acetate-acetic acid buffer (NaAc-HAc, pH 5.2). After removal of the unadsorbed fraction C1 with the same buffer, adsorbed proteins were eluted with 150 mM NaCl in the NaAc-HAc buffer to yield fraction C2 and then with 300 mM and 1 M NaCl in the NaAc-HAc buffer to yield fractions C3 and C4. Fraction C3 with hemagglutinating activity was then subjected to ion exchange chromatography on a 1.0 × 10 cm column of SP-Sepharose (GE Healthcare) in 10 mM NaAc-HAc buffer (pH 5.2). After removal of the unadsorbed fraction S1 with the same buffer, adsorbed proteins were eluted with a linear 0–0.5 M NaCl gradient in the NaAc-HAc buffer. The adsorbed fraction S3 was further purified by gel filtration on a fast protein liquid chromatography Superdex 75 10/300 GL column (GE Healthcare).

### 2.2. Determination of Molecular Weight and N-Terminal Sequence

The molecular weight of the active fraction (SU1) was subsequently determined by sodium dodecyl sulfate-polyacrylamide gel electrophoresis (SDS-PAGE) in accordance with the procedure of Laemmli and Favre [12]. Molecular weight determination was also performed by using FPLC-gel filtration as described above. N-terminal sequence determination of the protein was carried out using an HP G-1000A Edman degradation unit and an HP 1000 HPLC system [13].

### 2.3. Assay for Hemagglutinating Activity

A serial twofold dilution of the hemagglutinin solution in microtiter U-plates (25 *μ*L) was mixed with 25 *μ*L of a 2% suspension of rabbit red erythrocytes in phosphate-buffered saline (pH 7.2) at room temperature. The results were recorded after approximately half an hour when full sedimentation was observed in the blank. The hemagglutination titer, defined as the reciprocal of the highest dilution exhibiting hemagglutination, was regarded as one hemagglutination unit. Specific activity is the number of hemagglutination units/mg protein [2]. All determinations were performed in triplicate.

The hemagglutinating inhibition tests to investigate the inhibitory activities of various carbohydrates towards the hemagglutinin were performed in a manner analogous to the hemagglutination test. Serial twofold dilutions of sugar samples (200 mM to 0.195 mM) were prepared in phosphate-buffered saline. All of the dilutions were mixed with an equal volume (25 *μ*L) of a solution of the hemagglutinin with 32 hemagglutination units. The mixture was allowed to stand for 30 minutes at room temperature and then mixed with 50 *μ*L of a 2% rabbit erythrocyte suspension. The minimum concentration of the sugar in the final reaction mixture, which completely inhibited 32 hemagglutination units of the protein preparation, was calculated. The sugars tested included inositol, D-melibiose, D(−)-fructose, L(+)-rhamnose, sorbose, D(+)-galactose, D(+)-mannose, *α*-lactose, D(+)-xylose, L(+)-arabinose, D-glucose, maltose, raffinose, cellobiose, inulin, p-nitrophenyl-*α*-D-glucopyranoside, p-nitrophenyl-*β*-D-glucopyranoside, and inositol.

The effects of temperature, NaOH solution, HCl solution, and solutions of metallic chlorides (including those of Fe^2+^, K^+^, Ca^2+^, Cd^2+^, Cu^2+^, Hg^2+^, Mg^2+^, Mn^2+^, Pb^2+^, Zn^2+^, Al^3+^, and Fe^3+^) on hemagglutinating activity of the protein were examined as previously described [2].

### 2.4. Assay of Antiproliferative Activity on Tumor Cell Lines

The tumor cell lines Hep G2 (hepatoma) and L1210 (leukemia) were purchased from American Type Culture Collection (ATCC). The cell lines were maintained in Dulbecco modified Eagle's medium (DMEM) supplemented with 10% fetal bovine serum (FBS), 100 mg/L streptomycin, and 100 IU/mL penicillin at 37°C in a humidified atmosphere of 5% CO_2_. Cells (1 × 10^4^) in their exponential growth phase were seeded into each well of a 96-well culture plate (Nunc, Denmark) and incubated for 3 hours before addition of the hemagglutinin. Incubation was carried out for another 48 hours. Radioactive precursor, 1 *μ*Ci, ([^3^H-methyl] thymidine, from GE Healthcare) was then added to each well and incubated for 6 hours. The cultures were then harvested by using a cell harvester. The incorporated radioactivity was determined by liquid scintillation counting [14].

### 2.5. Assay for HIV-1 Reverse Transcriptase (HIV-1 RT) Inhibitory Activity

The assay for HIV-1 RT inhibitory activity was assessed by using an enzyme-linked immunosorbent assay (ELISA) kit from Boehringer Mannheim (Germany). The assay takes advantage of the ability of reverse transcriptase to synthesize DNA, starting from the template/primer hybrid poly(A) oligo(dT)_15_. The digoxigenin- and biotin-labeled nucleotides in an optimized ratio are incorporated into one of the same DNA molecules, which is freshly synthesized by the RT. The detection and quantification of synthesized DNA as a parameter for RT activity follow sandwich ELISA protocol. Biotin-labeled DNA binds to the surface of microtiter plate modules that have been precoated with streptavidin. An antibody to digoxigenin, conjugated to peroxidase (anti-DIG-POD), subsequently binds to the digoxigenin-labeled DNA. Finally, the peroxidase substrate is added. The peroxidase enzymes catalyze the cleavage of the substrate and produce a colored reaction product. The adsorbance of the samples at 405 nm can be determined by using a microtiter plate (ELISA) reader and is directly correlated to the level of RT activity. A fixed amount (4–6 ng) of recombinant HIV-1 RT was used. The inhibitory activity of the hemagglutinin was calculated as percentage inhibition as compared to a control without the protein [14].

### 2.6. Assay for Antifungal Activity

The assay for antifungal activity towards the phytopathogenic fungi* Fusarium oxysporum*,* Rhizoctonia cerealis*,* Rhizoctonia solani*, and* Sclerotinia sclerotiorum* was carried out in 100 × 15 mm petri dishes containing 10 mL of potato dextrose agar (PDA). After the mycelial colony had developed, sterile blank paper disks (0.625 cm in diameter) were placed at a distance of 0.5 cm away from the rim of the mycelial colony. An aliquot (15 *μ*L) of the hemagglutinin was added to a disk. The dishes were incubated at 23°C for 72 hours until mycelial growth had enveloped the disks containing the control and had formed crescents of inhibition around disks containing samples with antifungal activity [13].

## 3. Results

### 3.1. Purification of BSH

**Figure 1:**
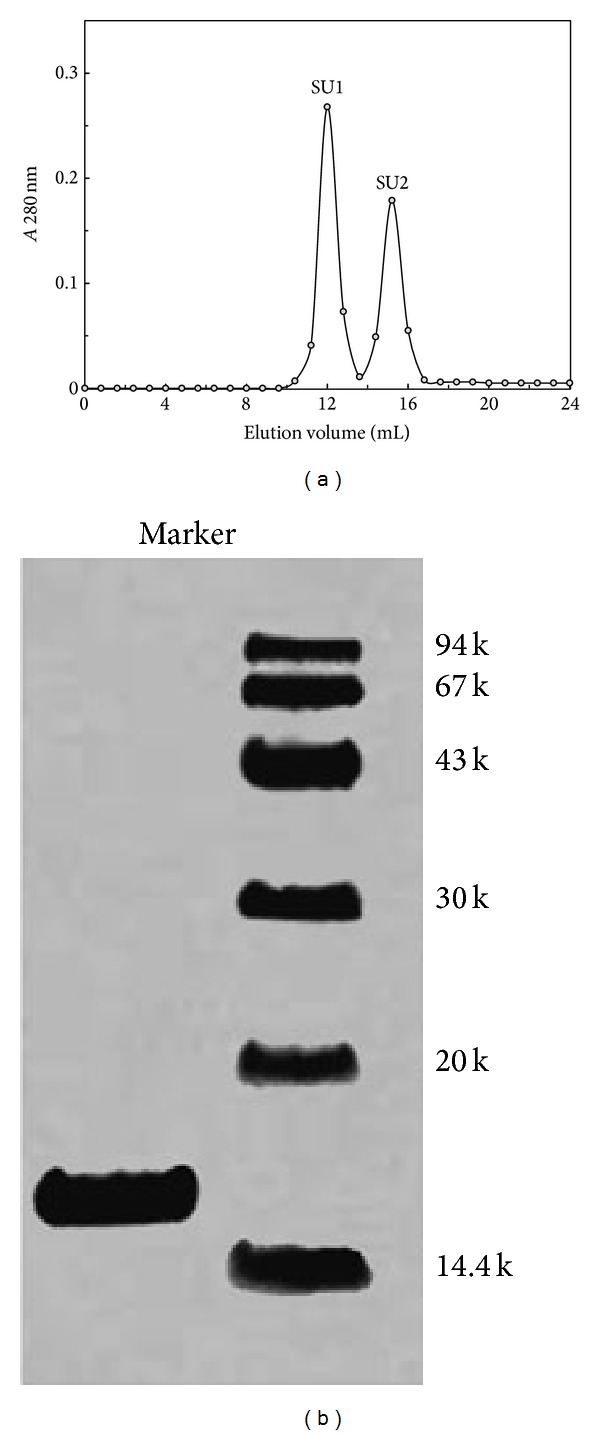
(a) Gel filtration of fraction on Superdex 75 HR 10/30 column by FPLC. Fraction SU1 represents purified hemagglutinin BSH. (b) SDS-PAGE of purified BSH. Right lane: molecular weight markers, from top downwards, phosphorylase b (94 kDa), bovine serum albumin (67 kDa), ovalbumin (43 kDa), carbonic anhydrase (30 kDa), soybean trypsin inhibitor (20 kDa), and lactalbumin (14.4 kDa).

The precipitate from ammonium sulfate precipitation was redissolved and dialyzed as crude protein extract. Then it was applied to an ion exchange chromatography Q-Sepharose column. One of the adsorbed fractions, fraction Q3, showed hemagglutinating activity (Table 1). Subsequently, the active fraction Q3 was applied to CM-Cellulose and yielded an unadsorbed fraction C1 and three adsorbed fractions C2, C3, and C4. Hemagglutinating activity resided in fraction C3 (Table 1). Upon ion exchange chromatography on SP-Sepharose, fraction C3 was resolved into a large unadsorbed fraction S1 and two smaller adsorbed fractions (S2 and S3) of approximately the same size. Activity resided in fraction S3. Upon gel filtration on Superdex 75, S3 was resolved into a larger peak SU1 (with a molecular mass of 31 kDa) and a smaller peak SU2 (Figure 1(a)). Hemagglutinating activity was enriched in SU1 which appeared as a single band with a molecular mass of 15.5 kDa in SDS-PAGE (Figure 1(b)). About 40-fold purification was achieved (Table 1).

### 3.2. Biological Activities of BSH

The interactions of lectins or hemagglutinins with cells can, in many instances, be inhibited specifically by simple sugars. However, BSH showed no specificity towards the various carbohydrates tested, including inositol, O-nitrophenyl-b-D-galactopyranoside, L-sorbose, raffinose, L-rhamnose, D-fructose, D-mannose, cellobiose, L-arabinose, D-xylose, D-melibiose, lactose, inulin, maltose, D-galactose, and D-glucose. It is suggested that the protein is a hemagglutinin but not a lectin. Its lower activities on human type O erythrocytes compared with type A, type B, and type AB indicate the binding is related to some ligand which has different distribution to blood types (Table 2).

The activity of BSH was stable up to 60°C followed by a precipitous decline from 64 to 8 hemagglutination units when the temperature was raised to 70°C. At and above 80°C activity was undetectable (Table 3). The hemagglutinin was stable in 6 mM HCl (pH 2.2) and 12.5 mM NaOH (pH 12.1), while it was reduced by 75% in 25 mM HCl (pH 1.3) and 100 mM NaOH (pH 13.0). The activity disappeared in 50 mM HCl (pH 1.9) and 200 mM NaOH (pH 13.3) (Table 3). The hemagglutinating activity was unaffected in the presence of K^+^, Cd^2+^, Cu^2+^, Mg^2+^, Mn^2+^, Zn^2+^, Al^3+^, and Fe^3+^ ions (1.25–10 mM) but was reduced by Fe^2+^ (5–10 mM), Ca^2+^ (5–10 mM), and Pb^2+^ (2.5–10 mM) and potently reduced by Hg^2+^(1.25–10 mM) ions (Table 4).

**Figure 2:**
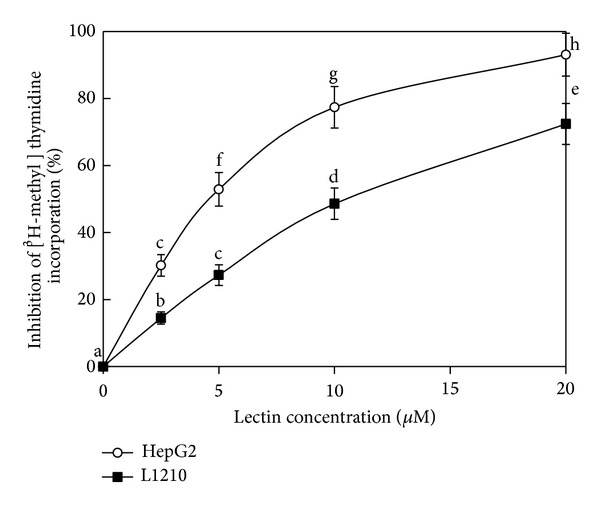
Antiproliferative activities of BSH towards Hep G2 and L1210 cell lines* in vitro*. The IC_50_ values towards Hep G2 and L1210 cells were 4.7 *μ*M and 7.0 *μ*M, respectively. Each value in both panels represents the means ± SD (*n* = 3). Different letters (a, b, c,…) next to the data points indicate statistically significant differences (*P* < 0.05) when the data were analyzed by analysis of variance followed by Duncan's multiple range tests.

**Figure 3:**
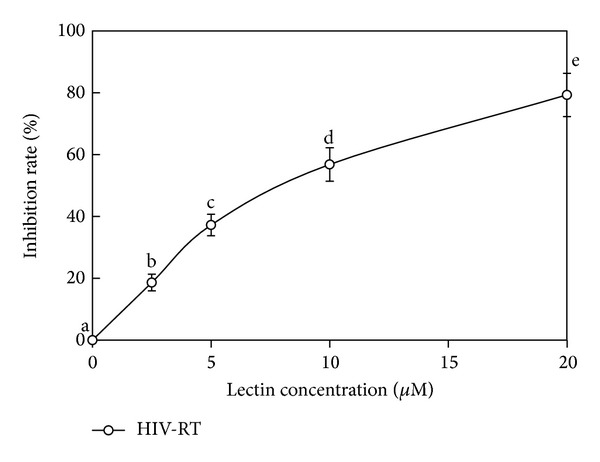
Inhibitory activity of BSH towards HIV-1 reverse transcriptase. It exhibited HIV-1 RT inhibitory activity with an IC_50_ of 7.1 *μ*M. Each value in both panels represents the means ± SD (*n* = 3). Different letters (a, b, c,…) next to the data points indicate statistically significant differences (*P* < 0.05) when the data were analyzed by analysis of variance followed by Duncan's multiple range tests.

BSH showed antiproliferative activity towards human hepatoma Hep G2 cells and mouse lymphocytic leukemia cells (L1210) with IC_50_ of 4.7 *μ*M and 7.0 *μ*M, respectively (Figure 2). It exhibited HIV-1 reverse transcriptase inhibitory activity with an IC_50_ of 7.1 *μ*M (Figure 3). No inhibitory activity against the test with four phytopathogenic fungi was found when the hemagglutinin amount was up to 2.5 mg (data not shown).

## 4. Discussion

Although mushroom species from genus* Boletus* are delicious (e.g.,* B. edulis*), some of them are toxic or even deadly.* B. magnificus* and* B. speciosus* cause hallucinogenic toxins and* B. erythropus* and* B. luridus* manifest insecticidal activities, while* B. satanas* causes deadly liver damage, gastrointestinal upset, and hemolysis [10, 15]. Very little is known about mushroom lectins or hemagglutinins from the genus* Boletus*, since they are rare in nature and cannot yet be artificially cultured.* B. speciosus* hemagglutinin (BSH) is compared herein with a lectin from another* Boletus* species,* B. edulis* lectin [11] (Table 5). The two blood cell hemagglutinating proteins share similar chromatographic behavior on cationic and anionic exchangers; both are adsorbed on CM-Cellulose and unadsorbed on DEAE-Cellulose. They are distinct from some other mushroom lectins which are adsorbed on both chromatographic media [16, 17]. Their molecular mass is around 32 kDa. They are both dimeric, which is in line with reports on dimeric lectins from* Agaricus edulis* [18],* Volvariella volvacea* [19],* Xerocomus spadiceus* [20], and* Lactarius flavidulus* [17]. Regarding thermostability, they can withstand temperatures no higher than 60°C. Nevertheless, they are more thermostable than lectins from some other mushrooms, like* Pleurotus ostreatus* [21],* Schizophyllum commune* [22], and* L. flavidulus* [17], which are unstable above 40°C. None of these mushroom lectins showed antifungal activity. However, BSH showed a lower pH stability and a higher HIV-1 RT inhibitory activity (IC_50_ was 7.1 *μ*M compared with 14.3 *μ*M for* B. edulis* lectin). The two* Boletus* hemagglutinating proteins demonstrate different sugar specificities.* B. edulis* lectin exhibited a unique sugar specificity towards xylose and melibiose, while BSH showed no specificity towards the variety of sugars tested. There are reports about lectins inhibited by fetuin and glycoprotein rather than simple saccharides [23]. The effects of various cations on the two proteins are also different. Fe^3+^ and Al^3+^ ions augmented the hemagglutinating activity of* B. edulis* lectin but none of the ions tested enhanced the hemagglutinating activity; Hg^2+^ ions potently inhibited the hemagglutinating activity of BSH but did not affect* B. edulis* lectin. Besides, the N-terminal sequence of BSH (ANVKIVK) exhibited no homology to* B. edulis* lectin (TYGIALRV) or any other purified fungal lectins.

Most of fungal lectins manifest antiproliferative activity against tumor cell lines. BSH is characterized by an ability to inhibit proliferation of two tumor cell lines, human hepatoma Hep G2 cells and mouse lymphocytic leukemia cells (L1210) with IC_50_ of 4.7 *μ*M and 7.0 *μ*M, respectively. Lectins from some other mushrooms, such as* Agaricus bisporus* (25 *μ*g/mL towards MCF-7 cells) [24],* Tricholoma mongolicum* (40 *μ*g/mL towards PU5-1.8 cells) [25],* V. volvacea* [19],* Pholiota adiposa* (2.1 *μ*M towards Hep G2 cells and 3.2 *μ*M towards MCF-7 cells) [26],* P. ostreatus* [21],* Russula lepida* (1.6 *μ*M towards Hep G2 cells and 0.9 *μ*M towards MCF-7 cells) [2],* L. flavidulus *(8.90 *μ*M towards Hep G2 cells and 6.81 *μ*M towards L1210 cells) [17], and* Hericium erinaceus* (56.1 *μ*M towards Hep G2 cells and 76.5 *μ*M towards MCF-7 cells) [27], also manifested this activity. The potent antiproliferative activity of BSH is remarkable and makes it a potential anticancer agent.

Screening of HIV reverse transcriptase inhibitors is currently a strategy to search for anti-HIV drugs since HIV RT is a key enzyme in the HIV life cycle [28]. BSH inhibited HIV-1 reverse transcriptase activity with an IC_50_ of 7.1 *μ*M. Other lectins, such as* L. flavidulus* lectin (IC_50_ of 5.68 *μ*M) [17],* P. adipose* lectin (IC_50_ of 1.9 *μ*M) [26], and* H. erinaceus* (IC_50_ of 31.7 *μ*M) [27], also exhibited anti-HIV-1 RT activity, while* R. lepida* lectin [2] lacked it. Inhibitors targeted to the HIV-1 RT can be classified into two groups: nucleoside RT inhibitors (NRTIs) and nonnucleoside RT inhibitors (NNRTIs) [29]. BSH, as a protein, belongs to NNRTIs group. The mechanism of inhibition is likely protein-protein interaction as in case of inhibition of HIV-1 reverse transcriptase by HIV-1 protease and cathelicidin [30]. Like lectins previously reported from mushrooms, BSH was devoid of antifungal activity [1]. Only a small number of the known constellations of plant lectins demonstrated antifungal activity [31].

## 5. Conclusion

In summary, a hemagglutinin with potent antiproliferative activity was isolated from thefruiting bodies of the hallucinogenic mushroom* B. speciosus*. It represents an addition to the existing list of mushroom hemagglutinating proteins since it is isolated from the family of rare mushrooms of* Boletus* genus and shows distinctive properties.

## Figures and Tables

**Table 1:** Yields and specific hemagglutinating activities of various chromatographic fractions at different stages of *B*. *speciosus* hemagglutinin (BSH) purification.

Fraction	Yield (mg)	Specific activity (U/mg)	Total activity (U)	Recovery of activity (%)	Folds of purification
Extract	1496.6	5.3	7904.0	100.0	1.0
Q3	176.4	30.4	5299.2	67.0	5.7
C3	35.9	88.7	3187.2	40.3	16.8
S3	10.5	193.0	2035.2	25.8	36.6
SU1	5.7	220.2	1255.1	15.9	41.7

**Table 2:** Hemagglutinating activity of BSH on rabbit and human erythrocytes.

	Rabbit erythrocytes	Human erythrocytes			
		A	B	O	AB
Hemagglutinating activity (%)	100	50	50	25	50

Note: initial hemagglutinating activity was 32 U.

**Table 3:** Effect of temperature and pH values on hemagglutinating activity of BSH.

Temperature (°C)	30	40	50	60	70	80
Residual hemagglutinating activity (%)	100	100	100	100	12.5	0

pH values	2.2	1.9	1.6	1.3	1.0	0.7
Residual hemagglutinating activity (%)	100	50	25	0	0	0

pH values	11.8	12.1	12.4	12.7	13.0	13.3
Residual hemagglutinating activity (%)	100	100	50	50	25	0

Note: initial hemagglutinating activity was 32 U.

**Table 4:** Effects of cations on hemagglutinating activity of BSH.

Cation (mM)	10	5	2.5	1.25
Hg^2+^	0	0	0	2
Fe^2+^	8	16	32	32
Ca^2+^	16	16	32	32
Pb^2+^	16	16	16	32
K^+^	32	32	32	32
Cd^2+^	32	32	32	32
Cu^2+^	32	32	32	32
Mg^2+^	32	32	32	32
Mn^2+^	32	32	32	32
Zn^2+^	32	32	32	32
Al^3+^	32	32	32	32
Fe^3+^	32	32	32	32

Note: initial hemagglutinating activity was 32 U.

**Table 5:** Comparison of characteristics of BSH with *B. edulis* lectin.

Characteristics	*Boletus speciosus* hemagglutinin	*B. edulis *lectin [11]
Chromatographic behavior		
DEAE ion exchanger	Unadsorbed	Unadsorbed
Q ion exchanger	Adsorbed	NA
SP ion exchanger	Adsorbed	NA
CM ion exchanger	Adsorbed	Adsorbed
Molecular mass (kDa)	31	32.6
Subunit molecular mass (kDa)	15.5	16.3
N-terminal amino acid sequence	ANVKIVK	TYGIALRV
Thermostability	60°C	60°C
pH stability	<12.5 mM NaOH; <6.25 mM HCl	<25 mM NaOH; <25 mM HCl
Sugar specificity	Unknown	Xylose, melibiose
Effect of cations on hemagglutinating activity	Inhibited by Hg^2+^, Fe^2+^, Ca^2+^, and Pb^2+*i*^ ions; no augmentation observed	inhibited by Mg^2+^, Mn^2+^, and Zn^2+^ ions; augmented by Fe^3+^ and Al^3+^ ions
Antifungal activity	Undetectable	Undetectable
Antiproliferative activity	IC_50_ of 4.7–7.0 *μ*M	NA
HIV-1 RT inhibitory activity	IC_50_ of 7.1 *μ*M	IC_50_ of 14.3 *μ*M

NA: not available.
